# Ultrasound-guided parasternal intercostal nerve block for postoperative analgesia in mediastinal mass resection by median sternotomy: a randomized, double-blind, placebo-controlled trial

**DOI:** 10.1186/s12871-021-01291-z

**Published:** 2021-03-31

**Authors:** Hexiang Chen, Wenqin Song, Wei Wang, Yawen Peng, Chunchun Zhai, Lihua Yao, Zhongyuan Xia

**Affiliations:** 1 Department of Anesthesiology, Renmin Hospital of Wuhan University, No. 99 Zhang Road, Wuhan, 430060 Hubei Province China; 2 Department of Psychiatry, Renmin Hospital of Wuhan University, No. 99 Zhang Road, Wuhan, 430060 Hubei Province China

**Keywords:** Ultrasound-guided parasternal intercostal nerve block, Postoperative analgesia, Mediastinal mass, Resection, Median sternotomy

## Abstract

**Background:**

Ultrasound-guided parasternal intercostal nerve block is rarely used for postoperative analgesia, and its value remains unclear. This study aimed to evaluate the effectiveness of ultrasound-guided parasternal intercostal nerve block for postoperative analgesia in patients undergoing median sternotomy for mediastinal mass resection.

**Methods:**

This randomized, double-blind, placebo-controlled trial performed in Renmin Hospital, Wuhan University, enrolled 41 participants aged 18–65 years. The patients scheduled for mediastinal mass resection by median sternotomy were randomly assigned were randomized into 2 groups, and preoperatively administered 2 injections of ropivacaine (PSI) and saline (control) groups, respectively, in the 3rd and 5th parasternal intercostal spaces with ultrasound-guided (USG) bilateral parasternal intercostal nerve block. Sufentanil via patient-controlled intravenous analgesia (PCIA) was administered to all participants postoperatively. Pain score, total sufentanil consumption, and postoperative adverse events were recorded within the first 24 h.

**Results:**

There were 20 and 21 patients in the PSI and control group, respectively. The PSI group required 20% less PCIA-sufentanil compared with the control group (54.05 ± 11.14 μg vs. 67.67 ± 8.92 μg, *P* < 0.001). In addition, pain numerical rating scale (NRS) scores were significantly lower in the PSI group compared with control patients, both at rest and upon coughing within 24 postoperative hours. Postoperative adverse events were generally reduced in the PSI group compared with controls.

**Conclusions:**

USG bilateral parasternal intercostal nerve block effectively reduces postoperative pain and adjuvant analgesic requirement, with good patient satisfaction, therefore constituting a good option for mediastinal mass resection by median sternotomy.

## Background

The mediastinum, central to the thoracic cavity, comprises the heart, trachea, thymus and esophagus; its structure is divided into 3 parts, including the anterior, middle and posterior mediastinum, from which multiple tumor types may originate [1]. Mediastinal masses comprise a broad range of tumors afflicting all age groups, constituting an important clinical challenge. Mediastinal space is narrow and the anterior mediastinum has the commonest mediastinal mass which is thymoma, followed by lymphoma [2].

Resection of a mediastinal mass by median sternotomy imparts substantial pain, which increases during movement and typically leads to chronic post-sternotomy pain as well as reduced pulmonary function caused by atelectasis and pneumonia [3, 4]. A multimodal analgesia regimen including opioids, nonopioid analgesics, such as nonsteroidal anti-inflammatory drugs (NSAIDs), antidepressants and local anesthetics (either via infusion or bolus doses), and various truncal blocks under ultrasound guidance are typical modalities used in combination to decrease postoperative pain to tolerable levels, enhance the recovery process, and reduce the need for opioid analgesia and the associated risk [5, 6].

**Fig. 1:**
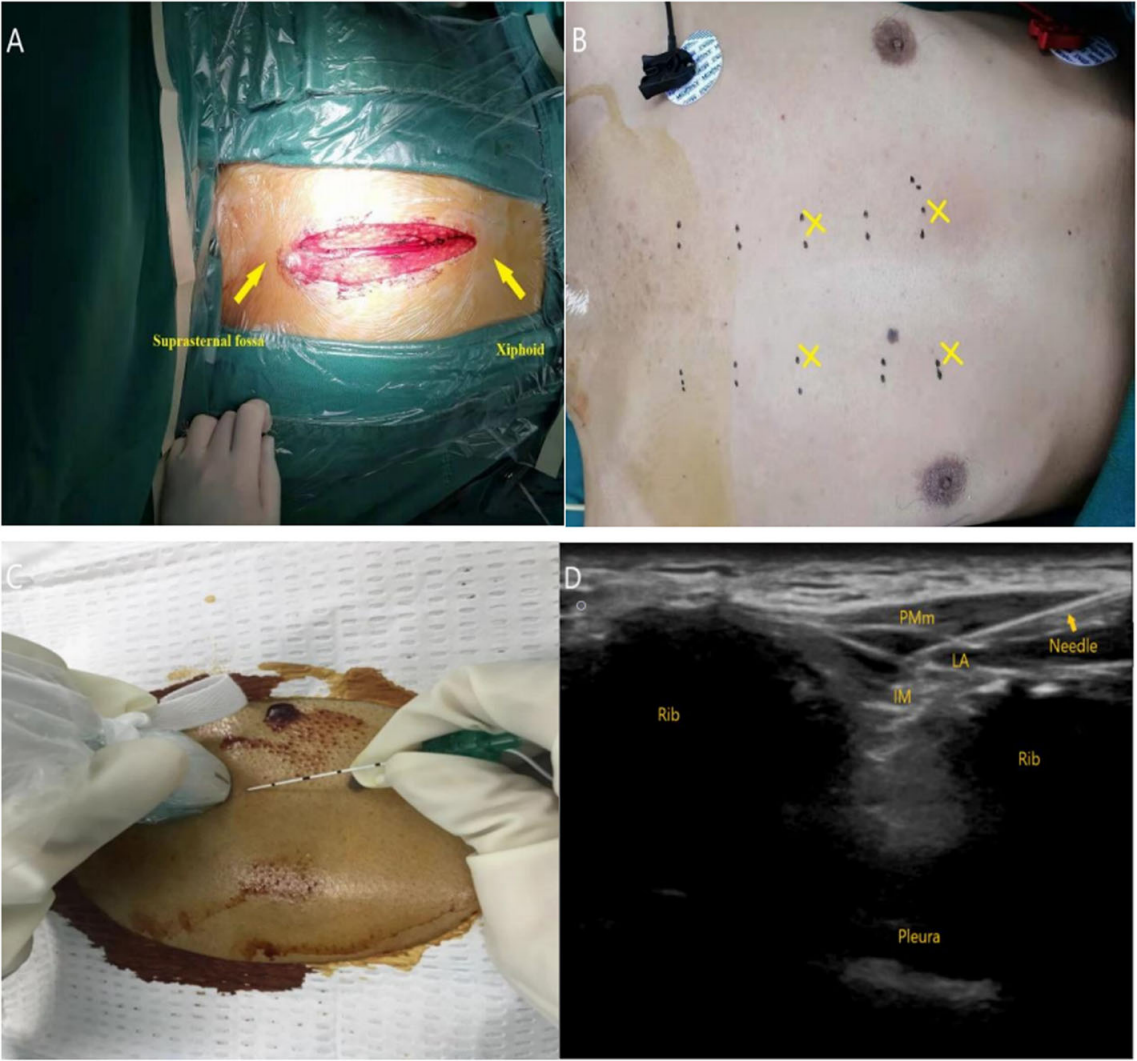
**a** Surgical incision of patients with median sternotomy for mediastinal mass resection. **b** Anatomic localization and puncture points. **c** Position of the ultrasound transducer and needle. **d** Ultrasound image of the puncture rout and the spread of solution for PSI block. PMm, pectoralis major muscle; IM, intercostal muscle; LA, local anesthetic

Parasternal intercostal nerve block (PSI) principally blocks anterior cutaneous intercostal nerves, and is used as an adjuvant for pain management post-cardiac surgery. This modality is highly effective in patients experiencing sternal wound pain following cardiac surgery [7–10]. However, USG parasternal intercostal nerve block in postoperative analgesia is rarely reported, especially in the context of thoracic surgeries involving median sternotomy for mediastinal mass resection [11–14]. As shown in Fig. 1a, the large surgical incision of patients with median sternotomy for mediastinal mass resection extended from suprasternal fossa to xiphoid.

Recently, we described a case administered USG bilateral parasternal block for perioperative analgesia during thymectomy via median sternotomy [15]. Based on this case report, we hypothesized that USG parasternal intercostal nerve block in mediastinal mass resection with median sternotomy is a useful tool in controlling postoperative pain and could reduce opioid requirements in patients receiving sufentanil patient-controlled intravenous analgesia (PCIA) during the first 24 h following surgery. To test this hypothesis, the present randomized, prospective, double-blind, placebo-controlled trial was carried.

## Methods

### Study design

This trial has been registered with the Chinese Clinical Trials Registry (Ref: ChiCTR1900026560). The protocols were designed in strict compliance to the principles of the Declaration of Helsinki, and approved by the Renmin Hospital of Wuhan University’s Ethics Committee. Each patient provided signed informed consent. Patient data and postoperative evaluations were double-blinded in this prospective, single-center, randomized, placebo-controlled study.

### Patients

Participants between 18 and 65 years old with American Society of Anesthesiologists (ASA) scores of I-II scheduled for elective mediastinal mass resection by median sternotomy between October 2018 and July 2019 were included. Exclusion criteria were: body mass index (BMI) > 35 kg/m^2^; renal failure; myasthenia gravis; mental disorder or inability to communicate; perioperative drug allergies; local infection in the area where the block was to be applied; technical errors in patient-controlled analgesia (PCA) devices (e.g., premature disconnection or dead battery).

### Randomization and blinding

Patients were then randomized based on a computer-generated random number table into 2 groups, including the ropivacaine (PSI) and saline (control) groups, respectively, preoperatively. All data collected (H.C.) and medication were double blinded, with both the patient and anesthesiologist providing the block (W.S.) and anesthesia management (W.W.) unaware of the pre-assigned groups. Unmasking did not come up until statistical analysis was achieved, which was supervised by Z.X.

### Intraoperative management

Upon arrival in the operating room, the patient was connected to a standard vital-signs monitor, which recorded blood oxygen saturation, heart rate, cardiac rhythm and non-invasive blood pressure. Surface electrodes were used to record the patient’s Narcotrend index with the Narcotrend Monitor (version 4.0; MonitorTechnik, Bad Bramstedt, Germany). All patients received infusion of lactated Ringer’s solution via a peripheral venous catheter. Patients of both groups were placed in the supine position and induced with general anesthetic agents including propofol at 1.5 to 2 mg/kg, sufentanil at 0.3 to 0.4 μg/kg and cisatracurium besilate at 0.2 to 0.3 mg/kg. Mechanical ventilation was performed to maintain oxygen saturation after tracheal intubation, with end-tidal carbon dioxide partial pressure kept at 35 to 45 mmHg.

USG bilateral parasternal intercostal nerve block with ropivacaine (PSI group) or saline (control group) was then performed. Ten minutes before the block, an anesthesiology trainee who was not part of the investigation prepared and handed over the ropivacaine or saline solutions packaged in identical bottles to the attending but blinded anesthesiologist.

### Nerve block procedure

Parasternal intercostal nerve block was carried out as previously described [11, 15–17]. Figure 1b shows anatomic localization and puncture points. The in-plane needle approach was applied under guidance of a high frequency linear-array ultrasound transducer. After standard chest skin disinfection, the transducer was covered with a sterile sleeve and placed transversely to the costal cartilage, parallel to the sternum. A 22-gauge 5-cm Tuohy needle was inserted 2 cm lateral to the midline and oriented between the pectoral major and external intercostal muscles in the 3rd parasternal intercostal space. Figure 1c shows position of the ultrasound transducer and needle. The prepared solutions (0.5% ropivacaine or 0.9% saline) were injected at 10 ml after withdrawing the needle without blood collection, and the spread of the injected solution and separation of the pectoralis major muscle from the rib and the external intercostal muscle confirmed the accuracy of the needle tip position. Figure 1d shows ultrasound image of the puncture rout and the spread of solution for PSI block. Ropivacaine or saline preparations were injected by the same method in the ipsilateral 5th parasternal intercostal space and the contralateral 3rd and 5th parasternal intercostal spaces, which a total of four blocks with 10 ml solution per point were performed. Considering the potential systemic toxicity of local anesthetics and the recommended maximal dose of 3 mg/kg for ropivacaine, low body weight patients were injected with 7–8 ml per point.

Anesthesia maintenance was performed with sevoflurane and remifentanil to keep the heart rate and mean arterial pressure within ±20% of respective baseline values and to maintain the Narcotrend index between 40 and 60. Cisatracurium and additional drugs were injected as needed. Warm Touch was used to keep the patient’s nasopharyngeal temperature between 36 °C and 37.5 °C. All patients underwent mediastinal mass resection by median sternotomy. At approximately 20 min prior to surgery end, intravenous injection of 0.1 μg/kg sufentanil, 50 mg flurbiprofen and 2 mg tropisetron were administered to all patients. Patients were extubated postoperatively if responding to verbal commands, with end-tidal carbon dioxide partial pressure below 45 mmHg and with a spontaneous respiratory rate exceeding 12 breaths/min.

### Pain assessment

A PCIA device (BCDB-200; BCM, Shanghai, China) was programmed to administer a continuous background infusion of 1.5 to 2.5 μg/h (< 55 kg, 1.5 μg/h; 55–75 kg, 2 μg/h; > 75 kg, 2.5 μg/h) and boluses of 1 μg sufentanil with 10-min lockout interval. The maximum dose of sufentanil allowed was 8 μg/h. The PCIA pump was maintained by an acute pain service team (Y.P., C.Z.).

Pain scores (0–10) at rest and upon coughing were recorded by the research staff (H.C.) using the numerical rating scale (NRS) [18] at 1, 3, 6, 12, and 24 h after surgery, respectively.

Rescue analgesia comprising intravenous flurbiprofen at 50 mg and tropisetron at 2 mg was provided in case of nausea or vomiting. The cumulative dose of sufentanil provided postoperatively within 24 h following surgery were collected.

Overall patient satisfaction (0, totally unsatisfied, 10, fully satisfied) scores were also recorded. Both groups were maintained on PCIA for at least 48 h after the procedure.

### Safety

Postoperative adverse events within 24 h were recorded in both groups, which including nausea, vomiting, bloating, dizziness, excessive sedation, and respiratory depression were assessed in both groups. Excessively sedated patients (defined as a Ramsay Sedation Scale [19] value of 5 or 6) were administered naloxone.

### Statistical analysis

Cumulative sufentanil use in the initial 24 h after surgery was the primary study outcome. Previous unpublished data in our setting between April and July 2018 revealed that approximately 70 ± 10 μg sufentanil is used in the initial 24 h post-median sternotomy for mass resection. A total of 20 patients per study group was the minimum needed to obtain a 95% power for detecting a 20% reduction in sufentanil requirements at an α value of 0.01. Therefore, we sought to enroll at least 25 patients in each study group to reduce the impact of potential patient exclusion due to missing data or dropout.

All study variables used descriptive statistics, and 2-tailed Student t test was performed to assess normally distributed data, while the non–normally distributed NRS data were depicted in box-and-whisker plots which each box extending from the upper quartile to the lower one. The final analysis utilized the Mann-Whitney U test for NRS data, including extremes, outliers and NRS score differences at the specified time points between the two groups. The χ2 or Fisher exact test was performed for categorical data. Data were presented as mean ± SD, mean (range), median (first and third quartiles), or percentage, as appropriate. *P* < 0.05 indicated statistical significance. The primary endpoints were assessed based on the modified intention-to-treat (ITT) population, i.e., excluding cases discharged before 24 h, with unplanned postoperative mechanical ventilation, and with a different surgical plan. SPSS version 18 (SPSS Inc.; Chicago, Illinois) was used for data analysis.

## Results

### Patient baseline features

**Fig. 2:**
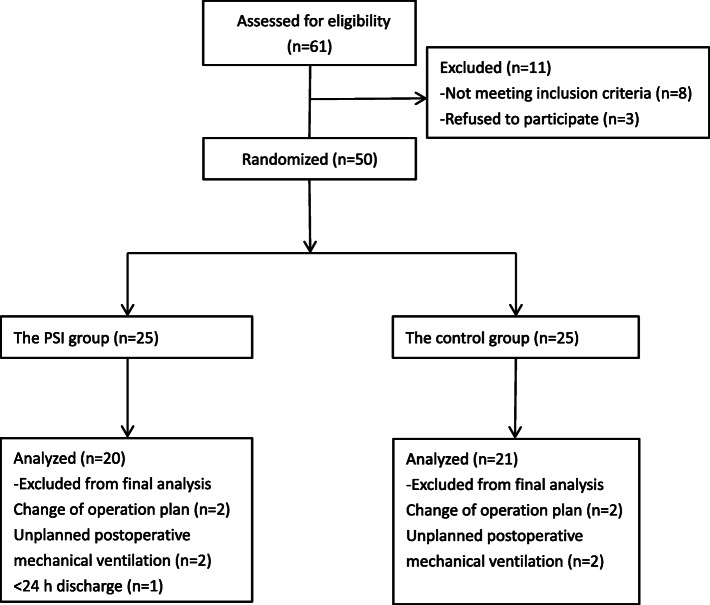
CONSORT study flow diagram

This study was performed confirming to Consolidated Standards of Reporting Trials (CONSORT) guidelines on parallel group randomized trails and the study flowchart is shown in Fig. 2 [20]. A total of 61 patients were enrolled, of which 8 failed to meet the inclusion criteria and 3 refused to participate. Another 9 patients were excluded due to discharge before 24 h after surgery (*n* = 1), unplanned postoperative mechanical ventilation (*n* = 4) and a different operative plan (*n* = 4). Finally, a total of 41 patients were assessed (modified ITT population), including 20 and 21 in the PSI and 21 control groups, respectively.

Table 1 summarizes the patient characteristics, durations of surgery and anesthesia, and intraoperative variables in the PSI and control groups. Both groups were similar in baseline characteristics and intraoperative variables.

**Table 1:** Clinicodemographic data

	PSI group (*n* = 20)	Control group (*n* = 21)	*P*-value
Gender (F/M)	11 / 9	9/ 12	0.44
Age (yr)	49.85 ± 11.80	43.90 ± 12.14	0.12
Weight (kg)	60.9 ± 11.46	66.60 ± 11.12	0.11
Height (cm)	162.60 ± 6.10	165.29 ± 6.75	0.19
ASA class (I/II)	8 / 12	13 / 8	0.16
Preoperative NRS	0.75 ± 0.44 (5.01)	0.62 ± 0.50	0.37
Duration of surgery (min)	118.50 ± 40.50	110.71 ± 27.99	0.48
Duration of anesthesia (min)	145.50 ± 40.84	137.62 ± 28.97	0.48
Total sufentanil (μg)	27.75 ± 4.72	28.57 ± 5.73	0.62
Total remifentanil (μg)	710.00 ± 228.61	704.76 ± 160.39	0.93
Operative blood loss (mL)	200.00 ± 79.47	200.00 ± 75.83	1.00
Intra-operative fluids (mL)	1677.50 ± 443.81	1514.29 ± 304.20	0.18
Urine output (mL)	545.00 ± 229.93	476.19 ± 210.13	0.32
Histopathological diagnosis (Thymoma/Lymphoma/Other)	13 / 3 / 4	13 / 4 / 4	0.94

Data are mean ± standard deviation or number (proportion) of patients

### Primary endpoints

Cumulative sufentanil consumption in the initial 24 h after surgery in the PSI group (54.05 ± 11.14 μg) was reduced by 20% compared with the control group (67.67 ± 8.92 μg, *P* < 0.001).

### Secondary endpoints

**Fig. 3:**
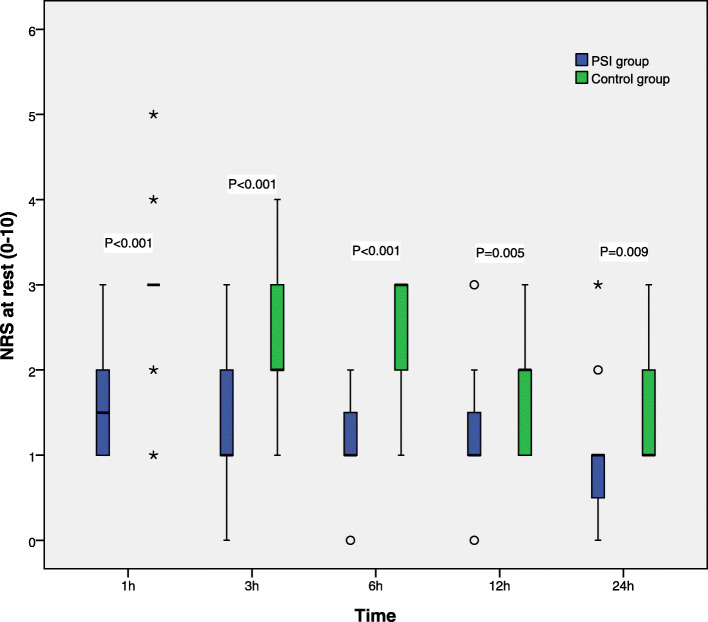
Resting pain scores in the first 24 h after surgery. NRS scores are shown in box-and-whisker plots, which contain the interquartile range (box), range not including outliers (error bars), and median (square in the box). Extremes (> 3 box lengths) and outliers (> 1.5 box lengths) are marked by asterisks and circles, respectively. Differences in NRS scores at various time points were analyzed by the Mann-Whitney U test. Resting pain scores at various time points were lower in the PSI group compared with control patients. NRS scores were 0 (no pain) to 10 (most severe pain)

**Fig. 4:**
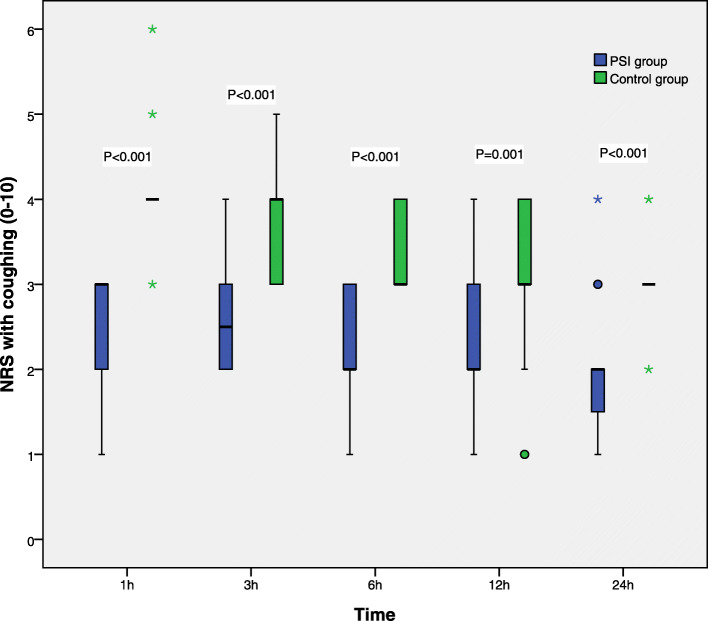
Coughing pain scores in the first 24 h after surgery. NRS scores are shown in box-and-whisker plots, which contain the interquartile range (box), range not including outliers (error bars), and median (square in the box). Extremes (> 3 box lengths) and outliers (> 1.5 box lengths) are marked by asterisks and circles, respectively. Differences in NRS scores at various time points were analyzed by the Mann-Whitney U test. Coughing pain scores at various time points were lower in the PSI group compared with control patients. NRS scores were 0 (no pain) to 10 (most severe pain)

Pain NRS scores at rest and upon coughing at 1, 3, 6, 12, and 24 h after surgery are displayed in Figs. 3 and 4, respectively, and were markedly higher in control patients compared with the PSI group at each time point (*P* < 0.05).

Requirement for rescue analgesia and patient satisfaction scores within 24 postoperative hours in both groups are shown in Table 2. Rescue analgesia within 24 h (flurbiprofen) was needed in 2 and 4 patients in the PSI and control groups, respectively. Patient satisfaction score of PSI group and control group was 8.20 ± 1.01 and 6.71 ± 0.64, respectively (*P* < 0.001).

**Table 2:** Rescue analgesia and patient satisfaction within 24 postoperative hours in the PSI and control Groups

	PSI group (*n* = 20)	Control group (*n* = 21)	*P*-value
Cumulative sufentanil consumption (μg) Patients requiring rescue analgesia within 24 h	54.05 ± 11.14	67.67 ± 8.92	< 0.001
Flurbiprofen axetil, n (%)	2 (10%)	4 (19%)	0.41
Satisfaction Score (0–10)	8.20 ± 1.01	6.71 ± 0.64	< 0.001

Data are mean ± standard deviation or number (proportion) of patients

### Adverse events

The postoperative adverse events within 24 h are shown in Table 3. A total of 3 and 7 patients in the PSI and control groups, respectively, experienced postoperative nausea. The incidence rates of vomiting, bloating, dizziness and excessive sedation were similar in both groups. Each group had one patient who experienced an episode of respiratory depression (respiratory rate < 8 breaths/min), which was reversed by lowering the background infusion levels of sufentanil. Patient satisfaction scores in the PSI and control groups were 8.20 ± 1.01 and 6.71 ± 0.64, respectively (*P* < 0.001).

**Table 3:** Adverse events

Postoperative complications 24 h	PSI group (*n* = 20)	Control group (*n* = 21)	*P*-value
Nausea, n (%)	3 (15%)	7 (33%)	0.17
Vomiting, n (%)	2 (10%)	3 (14%)	0.68
Bloating, n (%)	2 (10%)	3 (14%)	0.68
Dizziness, n (%)	1 (5%)	1 (5%)	0.97
Excessive sedation	0 (0%)	1 (5%)	0.32
Respiratory depression, n (%)	1 (5%)	1 (5%)	0.97

Data are mean ± standard deviation or number (proportion) of patients

## Discussion

This prospective, randomized, double-blind, placebo-controlled study showed that USG parasternal intercostal nerve block, following bolus initiation, reduces opioid requirements and adjunctive analgesia within the first 24 h after mediastinal mass resection by median sternotomy, with less postoperative adverse events. Patients administered USG parasternal intercostal nerve block reported significantly lower pain scores and higher satisfaction toward analgesia compared with those treated with sufentanil PCIA alone. Parasternal intercostal nerve block with ropivacaine has been successfully employed for pain management in cardiac surgery [7–10] and our previous study [15, 21], consistent with the present results. Regardless, evaluating the analgesic efficacy of USG parasternal intercostal nerve block with ropivacaine in patients undergoing mediastinal mass resection by median sternotomy was firstly performed in this study.

While mediastinal mass resection by thoracoscopy or robot-assisted surgery as a minimally invasive approach is widely used, median sternotomy as the traditional standard approach remains irreplaceable [22]. However, a significant adverse effect of the latter technique is the intense chest wall pain major originating from the median sternal wound. Meanwhile, ineffective postoperative pain management leads to reduced pulmonary function caused by atelectasis and pneumonia, coronary ischemia, poor wound healing, fatigue, insomnia, depression and the transition from acute pain to chronic pain [3, 4]. The intercostal nerve originates from the intervertebral foramen, branching into the lateral cutaneous branch and the anterior cutaneous branch at the mid-axillary line, which separates into the medial-branch that runs across the sternum and the lateral branch running in the breast tissue. Parasternal intercostal nerve block aims to neutralize anterior cutaneous intercostal nerves with the goal of reducing sternal nociception which may exert preemptive analgesic effects, and restraining the establishment of altered central processing of afferent input which may amplify postoperative pain [13, 14]. This technique is considered a good modality for adjuvant analgesia. However, parasternal intercostal nerve block does not neutralize the lateral intercostal nerves or other somatic and visceral nerves, both of which are sources of musculoskeletal nociceptive pain in the chest wall area post-median sternotomy [11, 23].

Adjuvant nonsteroidal anti-inflammatory drugs are often employed to reduce opioid requirements [6]. Opioids used as the sole agent carry risks of excessive sedation, respiratory depression and gastrointestinal reaction [24, 25]. This study adopted a multimodal analgesia regimen, including opioids, nonopioid analgesics and a nerve block for optimal pain relief, and was successful in achieving good patient satisfaction with low postoperative adverse events. The innervation of mediastinum is autonomic and sympathetic nervous system including phrenic and intercostal nerves as well as the esophageal plexus, which the PCIA could provide intense analgesia for this nociceptive pain. Meanwhile, the bilateral and multi-segmental anterior cutaneous intercostal nerves blockade could generate valid pain relief for the skin, muscle and periosteum of sternum at the site of surgical incision. Finally, the cumulative opioids consumption and adverse events were reduced. The USG parasternal intercostal nerve block averted the negative side effects observed with other postoperative analgesia modalities, and epidural analgesia and paravertebral block, in cardiac surgery patients. Furthermore, these modalities are operator-dependent and require experienced professionals for safe and quick application [26–28]. In this study, parasternal intercostal nerve block under real-time ultrasound guidance was performed near the sternum by injecting ropivacaine between the external intercostal and pectoralis major muscles, resulting in a larger area of sensory deprivation in comparison with transversus thoracic muscle plane block [29]. In the latter approach, the injected analgesic flows anteriorly to the transversus thoracic muscle, making USG application difficult. Anatomical experiments have found that the plane between the pectoralis major muscle and the external intercostal muscle is the same as that between the pectoralis major muscle and the ribs because the pectoralis minor muscle originates from the middle of the third [30], fourth and fifth ribs, while the hard ribs act as fences to prevent the needle from going deeper. Therefore, we implemented parasternal intercostal nerve block only in the 3rd and 5th parasternal intercostal spaces, which would result in 2nd to 6th parasternal intercostal nerve block after diffusion. Meanwhile, there were no technique- or drug-related complications such as pneumothorax and deep sternal wound infection invading the mediastinum, muscle and bone in this study at 1 week after surgery. We also believe that USG parasternal intercostal nerve block may be applied for chest wall keloid scar resection.

This study had several limitations. Firstly, a short-term follow up of patients was performed, limiting the ability to assess long-term chest wall pain. Although patients administered USG parasternal intercostal nerve block had lower pain scores, formal sensory block assays were not carried out. Meanwhile the significant reduction in opioid use in the PSI group is a strong indication that analgesia was present. Paravertebral block with 0.5% ropivacaine or 0.5% bupivacaine imparts pain relief lasting between 6 and 8 h post-thoracoscopy [31]. However, this time frame was not assessed in this study, whose goal was to evaluate the reduction in adjuvant opioid use. In addition, it remains unclear whether reduced adjunctive analgesia and opioid requirements in the PSI group compared with control patients within the first 24 h after surgery are only associated with the early postoperative period. USG parasternal intercostal nerve block may not be ideal in the ward setting and in case of more extensive surgical incisions, for which continuously infused local anesthetics for postoperative analgesia after median sternotomy is more advantageous [32]. Therefore, modifications to the continuous parasternal intercostal nerve block protocol for pain management warrant further investigation. Given the limited demographic profile of the study population, additional larger investigations are required to confirm these findings, thereby advocating this technique for application in the general population.

## Conclusions

Ultrasound-guided parasternal intercostal nerve block as a simple and practical technique is effective in producing an opioid-sparing effect, and results in less postoperative pain in mediastinal mass resection by median sternotomy.

## Data Availability

The datasets used and/or analysed during the current study are available from the corresponding author on reasonable request.

## Abbreviations

USG: Ultrasound-guided
PSI: Parasternal intercostal nerve block
PCIA: Patient-controlled intravenous analgesia
NSAIDs: Nonsteroidal anti-inflammatory drugs
PCA: Patient-controlled analgesia
